# NiFe on CeO_2_, TiO_2_, and ZrO_2_ Supports as Efficient Oxygen Evolution Reaction Catalysts in Alkaline Media

**DOI:** 10.1021/acsaem.4c03268

**Published:** 2025-02-24

**Authors:** Neethu Kochukunnel Varghese, Elina Mkrtchian, Anshika Singh, Letizia Savio, Massimiliano Boccia, Vincenza Marzocchi, Antonio Comite

**Affiliations:** † Department of Chemistry and Industrial Chemistry, University of Genoa, 16146 Genoa, Italy; ‡ IMEM-CNR UOS Genoa, 16146 Genoa, Italy; § Department of Physics, University of Genoa, 16146 Genoa, Italy; ∥ H2 Energy SRL, 26026 Pizzighettone, Cremona, Italy

**Keywords:** NiFe, oxygen evolution reaction, electrocatalysts, AEM ionomer, alkaline electrolysis, green hydrogen

## Abstract

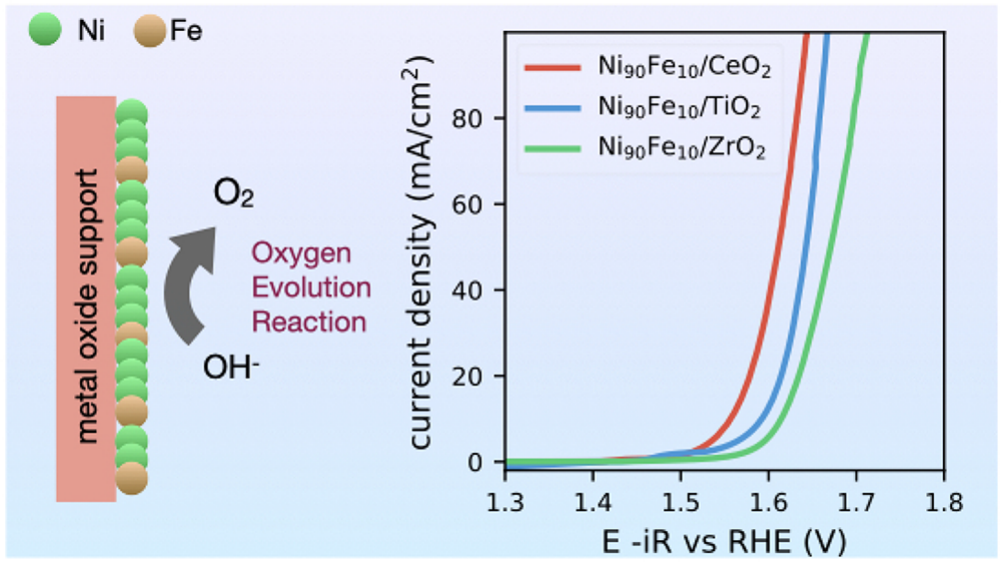

The high cost and low energy efficiency of conventional water electrolysis methods continue to restrict the widespread adoption of green hydrogen. Anion exchange membrane (AEM) water electrolysis is a promising technology that can produce hydrogen using cost-effective transition-metal catalysts at high energy efficiency. Herein, we investigate the catalytic activity of nickel and iron nanoparticles dispersed on metal-oxide supports for the oxygen evolution reaction (OER), employing electrochemical testing with an anion exchange ionomer to evaluate their potential for application in AEM electrolyzers. We report the electrochemical performance of NiFe nanoparticles of varying Ni:Fe ratios on CeO_2_ for OER reaction, assessing the overpotential, Tafel slope, and electrochemical stability of the catalysts. Our findings indicate that Ni_90_Fe_10_ has the highest catalytic activity as well as stability. To further understand the role of different supports, we assess the electrocatalytic performance of Ni_90_Fe_10_ nanoparticles on two more supports - TiO_2_ and ZrO_2_. While CeO_2_ has the lowest overpotential, the other supports also show high activity and good performance at high current densities. TiO_2_ exhibits superior stability and its overpotential after chronopotentiometry measurements approaches that of CeO_2_ at high current densities. These results underscore the critical role of iron addition in enhancing nickel nanoparticles’ catalytic activity and further emphasize the importance of metal oxide supports in improving catalyst stability and performance.

## Introduction

Water electrolysis for green hydrogen production is a promising technology to harness intermittent renewable energy for the production of high-value chemicals and fuels.^1−4^ Anion exchange membrane (AEM) alkaline water electrolysis is an emerging technology that combines the advantages of proton exchange membrane (PEM) water electrolyzers and traditional alkaline water electrolyzers.^5−10^ The main bottleneck in the water-splitting reaction in alkaline media is the oxygen evolution reaction (OER) due to its slow kinetics. Thus, to enhance the efficiency of AEM electrolyzer technology, developing more effective OER catalysts compatible with anion exchange membranes is crucial.^6,11^

The OER reaction in an alkaline medium is commonly assumed to proceed through the adsorbate evolution mechanism, where the reaction pathway includes the oxidation of 4 OH^–^ ions and the release of 4 electrons, leading to slow reaction kinetics.^6,12^ The exact reaction pathway and mechanism can vary depending on the catalyst’s structure, as different catalysts can preferentially stabilize reaction intermediates.^13,14^ Therefore, optimizing the catalyst structure could be critical in improving the OER kinetics.^12,13,15^

Over the years, research has focused on nickel and iron as promising anode catalysts for the OER under alkaline conditions due to their cost-effectiveness, high catalytic activity, and stability.^16−22^ Furthermore, the addition of Fe to Ni-based catalysts has been shown to increase their electrocatalytic activity.^19,23,24^ It is also known that catalyst supports play a critical role in improving electrocatalytic activity and stability by maintaining a uniform dispersion of the particles on the surface of the support, enhancing the atomic utilization, preventing agglomeration, and in turn stabilizing the active sites.^25−27^ For the OER, noncarbon supports such as metal oxides, nitrides, and metal carbides have shown excellent stability and metal–support interactions even in the harsh oxidizing environment.^28^

While NiFe catalysts are well established for their effectiveness in alkaline OER, much of our understanding is derived from studies employing PEM ionomers such as Nafion. However, ionomers are known to influence catalyst layer morphology and reactant transport,^29^ and their ionic conductivity and stability can also impact catalytic activity.^29^ Given the increasing importance of AEM electrolyzers, it is crucial to assess the effect of AEM ionomers on the catalyst performance. Notably, NiFe catalysts exhibit low electrochemical activity when used with Aemion+ (an AEM ionomer), as it suppresses the Ni^2+^ to Ni^3+^ transformation.^30^ This raises an important question: can the electrocatalytic performance of NiFe catalysts in the presence of an AEM ionomer be improved, potentially through the use of support?

Among supports, CeO_2_, with its beneficial oxygen storage capacity and redox properties, has shown a potential to enhance catalytic activity.^31,32^ However, within an AEM system, such as Aemion+, the performance of NiFe on CeO_2_ remains poorly understood. Another related consideration is a systematic comparison of NiFe catalysts with different metal oxide supports. Different supports offer unique characteristics that could impact catalyst stability and OER activity, and understanding these differences could guide the selection of optimal supports for AEM electrolyzers. The activity of the metal sites can vary depending on the metal–support interactions and the ability of the oxide to stabilize the metal nanoparticles.

An additional parameter that can also affect the catalyst performance is the nature of the metal oxide precursors. Colloidal metal oxide precursors may offer distinct advantages over commonly used powder precursors in synthesis, such as improved metal dispersion, which enhances the reaction rates and catalyst stability. In solution, the oxide support helps to stabilize the metal precipitates by lowering the surface energy, thereby reducing the nucleation energy barrier. This effect allows the support surface to act as a seed for nucleation. Additionally, the high surface area provided by colloidal dispersion can promote smaller particle sizes for the precipitated metal atoms, leading to better catalytic performance and stability in OER applications.^33^

In this work, we investigate the catalytic performance of NiFe on metal oxide supports combined with an AEM ionomer by exploring the effect of metal–support interactions and the Fe load. Using a colloidal suspension of CeO_2_, TiO_2_, or ZrO_2_ nanoparticles, we achieve improved uniformity of NiFe nanoparticles on the oxide surfaces. We used a commercial anion exchange ionomer (Aemion+) to prepare the catalyst ink. Our electrochemical characterization using a rotating disk electrode (RDE) setup shows that, when integrated with an AEM ionomer, the Ni_90_Fe_10_ composition exhibits the best overall OER performance on the CeO_2_ support. Interestingly, the catalytic performance lies in a nonconvex manifold when the Fe ratio is varied, with multiple local minima, indicating a complex relationship between Fe content and OER efficiency. We apply the optimal Ni_90_Fe_10_ ratio to TiO_2_ and ZrO_2_ supports to further evaluate the influence of different oxides on the OER kinetics, providing insights into support-dependent performance in alkaline environments.

## Materials and Methods

### Catalyst Synthesis

NiFe/CeO_2_ catalysts were synthesized by chemically reducing the metal salts onto the CeO_2_ surface using sodium borohydride (NaBH_4_) as a reducing agent. The chemical reduction of Ni and Fe nanoparticles on the surface of CeO_2_ followed the reaction. Ni(NO_3_)_2_·6H_2_O + Fe(NO_3_)_3_·9H_2_O + NaBH_4_ + H_2_O → NiFeB + NaNO_3_ + H_2_ + B(OH)_3_. The catalysts were synthesized in Ni:Fe ratios of 1:0, 0.9:0.1, 0.75:0.25, 0.5:0.5, 0.25:0.75, 0.1:0.9, and 0:1 using 5 mmol of total metal precursors in 25 mL of ethanol. The metal precursors were nickel(II) nitrate hexahydrate (Ni(NO_3_)_2_·6H_2_O, Thermo Scientific, ≥98%) and iron(III) nitrate nonahydrate (Fe(NO_3_)_3_·9H_2_O, Sigma-Aldrich, ≥98%). The metal precursors were stirred for 10 min at 600 rpm in a magnetic stirrer until they completely dissolved. 30 wt % equivalent CeO_2_ as a colloidal solution (20% in H_2_O, 10–20 nm, Alfa Aesar) was added to the mixture and stirred for 30 min at 600 rpm to obtain a uniform distribution of the colloidal particles. 12 mmol of sodium borohydride (NaBH_4_, Sigma-Aldrich, 99%) was dissolved in 15 mL of water and then added to the reaction mixture slowly and further stirred for 30 min. The solution turned black, and bubble formation was observed during the reduction. The catalysts were washed with ethanol 3 times using a centrifuge at 4000 rpm for 10 min. The catalysts were then oven-dried for 12 h at 60 °C. Ni_90_Fe_10_/ZrO_2_ and Ni_90_Fe_10_/TiO_2_ were also synthesized following a similar procedure by using colloidal ZrO_2_ (20% in H_2_O, 5–10 nm, Alfa Aesar) or TiO_2_ (20–35% in H_2_O, Alfa Aesar) instead of CeO_2_.

### Physical and Chemical Characterization

The morphology and the composition of the catalysts were observed using field emission-scanning electron microscopy (FE-SEM, Zeiss SUPRA 40 VP), high-resolution transmission electron microscopy (HR-TEM, JEOL 2100 Plus) equipped with STEM (HAADF and BF) detectors, and energy-dispersive X-ray spectroscopy probe (EDS, window 100 mm^2^, Bruker Flash 6T) detector. The Brunauer–Emmett–Teller (BET) surface areas of the catalysts were assessed through N_2_ adsorption–desorption experiments at 77 K using the ASAP 2020 physisorption analyzer (Micrometrics Instrument Corporation). X-ray diffraction (XRD, Rigaku SmartLab X-ray diffractometer) analysis was conducted using a Cu Kα radiation source (λ = 0.154 nm), operating at 40 kV and 150 mA, to understand the crystalline properties of the catalysts. Silicon zero background disks were used as sample holders. The samples were washed five times with water before XRD measurements to remove residual NaNO_3_ from the synthesis.

Surface composition was analyzed by X-ray photoemission spectroscopy (XPS), using a hemispherical analyzer (Model 10-360, Physical Electronics) coupled to a monochromatic X-ray source (Model 10-610, Physical Electronics). Measurements were conducted in an ultrahigh vacuum chamber with base pressure below 5 × 10^–8^ mbar, equipped with a load-lock system for rapid sample introduction. Monochromatic Al Kα radiation (*h*ν = 1486.6 eV) was used to excite the photoelectrons, which were collected from a spot approximately 100 μm in diameter. An electron gun was employed to maintain charge neutrality during measurement. Spectral analysis was performed using KolXPD software. The binding energies (*E*_b_) were calibrated by setting the C 1s peak from the adventitious carbon to 284.4 eV, following established protocols.^34,35^ For consistent comparison, in each region, all traces were normalized to the low-binding-energy side. The C 1s, O 1s, and Ni 3p regions were fitted using Voigt functions after subtracting a Shirley background, while Voigt doublets were used to fit the Ni 2p region.

### Electrochemical Characterization

Electrochemical characterization was conducted in a three-electrode cell setup with rotating disc electrode (RDE) measurements following the protocol from ref (36) (Biologic VMP3 Potentiostat and BluRev Rotating disc electrode). A Pt wire was used as the counter electrode, and a Hg/HgO electrode (Sigma-Aldrich) was used as the reference electrode. The working electrode was a 5 mm diameter glassy carbon electrode (Biologic) and the working electrode was polished with 0.5 μm alumina (Ossila) on polishing pads before coating the catalysts.

The catalyst ink was prepared by dispersing 30 mg of the catalyst in 2.97 g of ethanol. An Aemion+ anion exchange ionomer solution (2 wt % in a 1:1 ethanol:acetone mixture, Ionomr Innovations) was added, maintaining an ionomer-to-catalyst ratio of 0.1. The catalyst ink was then sonicated with a probe sonicator for 5 min using 2 s pulses of 40% amplitude. Eight microliters of the catalyst ink were drop-casted on the glassy carbon electrode and were kept overnight for drying. The OER catalyst loading was 0.4 mg/cm^2^ for all of the catalysts. All electrochemical measurements were done using N_2_ saturated 1 M KOH electrolyte at room temperature (20 ± 2 °C). The electrolyte was purged with N_2_ gas to remove any dissolved gases during electrochemical measurements.

Each catalyst was conditioned for 50 cycles to ensure that it was stable and in a reproducible state. The conditioning was done between 1.00 and 1.45 V versus the reversible hydrogen electrode (RHE) with a scan rate of 100 mV/s in a stagnant electrolyte. The activity was measured using linear sweep voltammetry (LSV) measurements from 1.00 V vs RHE to 1.80 V vs RHE at 1600 rpm at a scan rate of 5 mV/s. The LSV measurements were crosschecked using stationary polarization measurements where the potential of the electrode is held constant for 60 s at 1.00, 1.25, 1.50, 1.55, 1.60, 1.65, 1.70, 1.75, and 1.80 V vs RHE while the electrode rotates at 1600 rpm. To investigate the electrochemical stability of the catalysts, chronopotentiometry measurements were conducted at a current density of 10 mA/cm^2^ (geometric area) for 2 h at 1600 rpm. Activity was measured after the stability test as well. The system was brought to equilibrium between each activity and stability measurement by measuring the open circuit potential for 600 s. Electrochemical impedance spectroscopy (EIS) was done at an amplitude of 10 mV root-mean-square (rms) alternating current (AC) perturbation in a 10^–1^–10^5^ Hz frequency range at a constant potential 1.6 V vs RHE. The Ohmic resistance was compensated at 100% of the high-frequency resistance at 1.6 V vs RHE during data processing.

The potential with respect to RHE in 1 M KOH was calculated using the following equation:^37^
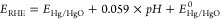
where *E*_Hg/HgO_^0^ = 0.098 V. The overpotential (η) was calculated as follows:
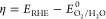
where *E*_O_2_/H_2_O_^0^ = 1.23 V.^38^ The electrochemical testing of Ni_90_Fe_10_/ZrO_2_, and Ni_90_Fe_10_/TiO_2_ was done with the same procedure as described above. The only difference was the use of graphite as the counter electrode and the use of RDE from Pine Instruments. The cyclic voltammetry (CV) measurements for the determination of electrochemical surface area (ECSA) were done in the static solution in the non-Faradaic current range from 1.0 V vs RHE to 1.3 V vs RHE at 7 different scan rates: 10, 20, 50, 100, 200, 300, and 500 mV/s. The double-layer capacitance of the catalysts was obtained from the slope of the linear fit of the current versus scan rate in a non-Faradaic potential range.^39^ Details of the uncertainty estimation are provided in the Supporting Information.

## Results and Discussion

### Physical and Chemical Characterization

**Figure 1:**
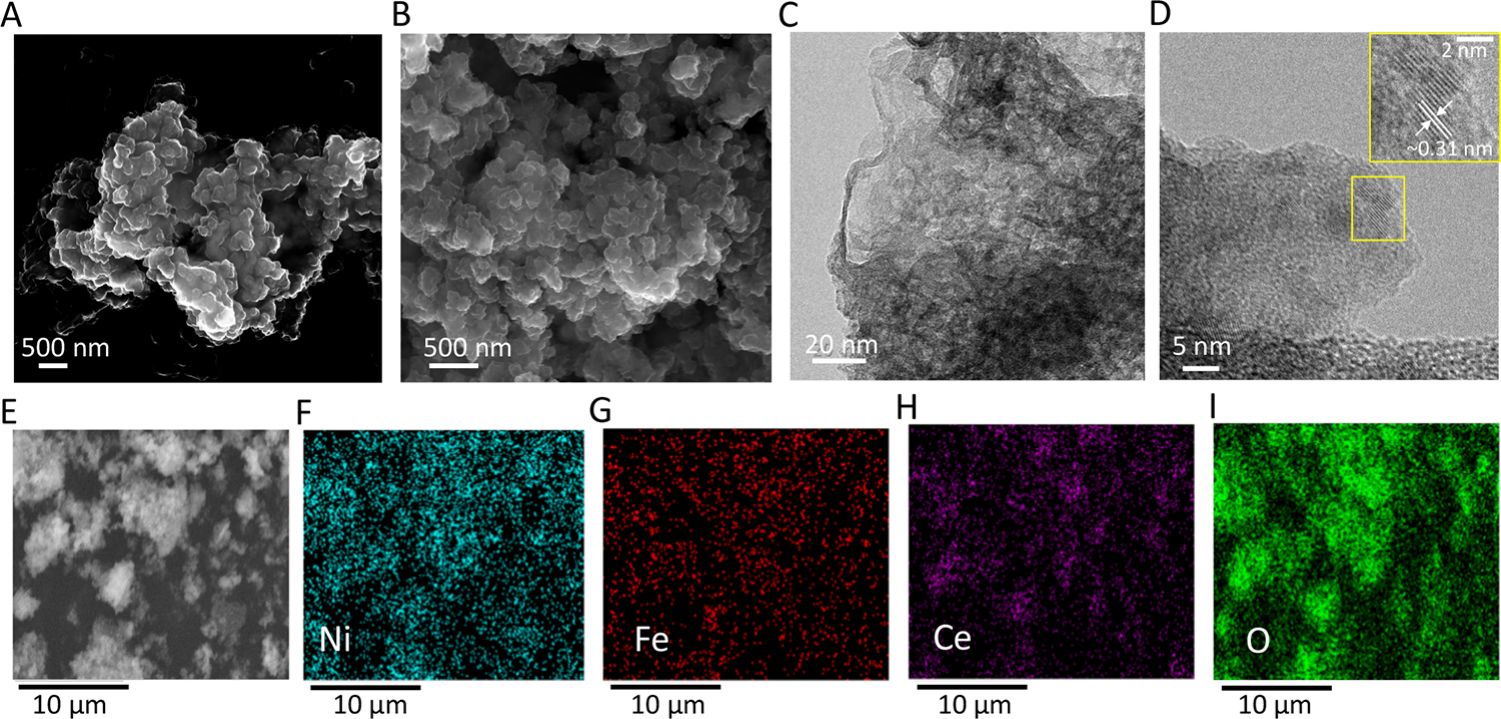
Electron micrographs of the Ni_90_Fe_10_/CeO_2_ catalyst. (A,B) Field emission-scanning electron microscopy (FE-SEM) images of the *as-prepared* Ni_90_Fe_10_/CeO_2_ catalyst. (C) Transmission electron microscopy (TEM) images and (D) high-resolution TEM (HR-TEM) images of the same catalyst. (E) SEM image of the area analyzed in panels (F–I) using SEM-energy dispersive X-ray spectroscopy (EDS), showing the elemental distribution of (F) Ni, (G) Fe, (H) Ce, and (I) O.

FE-SEM, TEM, and HR-TEM images show that the morphology of the Ni_90_Fe_10_/CeO_2_ catalyst is that of stacked nanosheets with Ni and Fe nanoparticles dispersed on the surface of CeO_2_ (Figure 1A–D). We observe lattice fringes with an interlayer spacing of ∼0.31 nm (Figure 1D) and ∼0.7 nm (Figure S2B) corresponding to the (111) planes of CeO_2_^40^ and (003) planes of α-Ni(OH)_2_^41^, respectively. All catalysts in this study exhibited similar morphology (Figure S1). Ni, Fe, Ce, and O elements show a homogeneous distribution over the catalyst surface, as revealed through the SEM-EDS spectra (Figure 1E–I) and HAADF STEM-EDS spectra (Figure S2D–G). The homogeneous dispersion of the Ni and Fe nanoparticles enhances the active surface area of the catalyst, and this nanostructured configuration promotes the OER reaction through efficient electron transfer and by improving the conductivity of the CeO_2_ support. Flame atomic absorption spectroscopy (FAAS) measurements for quantitative elemental analysis indicate that the experimental Ni:Fe ratios are in good agreement with the desired ratios (Table S1). Thus, chemical reduction using NaBH_4_ is not only a fast but also an efficient and accurate method for metal doping.

**Figure 2:**
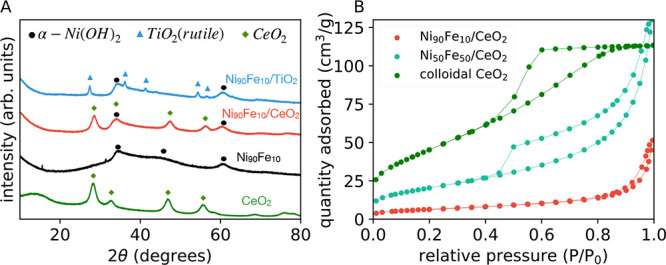
(A) X-ray diffraction (XRD) spectra of Ni_90_Fe_10_/TiO_2_, Ni_90_Fe_10_/CeO_2_, Ni_90_Fe_10_ and CeO_2_. (B) Brunauer–Emmett–Teller (BET) isotherm measurements of colloidal CeO_2_, Ni_50_Fe_50_/CeO_2_ and Ni_90_Fe_10_/CeO_2_.

The XRD spectrum of pure CeO_2_ exhibits distinct peaks at 2θ values of 28.53, 33.08, 47.5, and 56.35 corresponding to the (111), (200), (220), and (311) planes of cubic CeO_2_ used as a support (Figure 2A). These characteristic peaks are also observed in the Ni_90_Fe_10_/CeO_2_ sample, confirming the presence of cubic CeO_2_. For the Ni_90_Fe_10_/TiO_2_ sample, XRD peaks at 2θ values of 27.4, 36.2, 41.2, 43.9, 54.3, and 56.5 correspond to the (110), (101), (200), (111), (210), (211), and (220) planes of the rutile TiO_2_ structure. The presence of TiO_2_ and CeO_2_ confirms that the Ni and Fe nanoparticles are successfully supported on oxide matrices, thereby providing a stable heterostructure.

The unsupported Ni_90_Fe_10_ sample shows peaks at 2θ values of 35.5 and 60.4. These peaks can be attributed to (101) and (110) planes of α-Ni(OH)_2_ (JCPDS No. 38-0175).^42,43^ These Ni(OH)_2_ peaks are also present in the Ni_90_Fe_10_/CeO_2_ and Ni_90_Fe_10_/TiO_2_ samples. It is important to note that α-Ni(OH)_2_ has a less crystalline order compared to β-Ni(OH)_2_.^44^ Thus, the disordered α phase is particularly advantageous as it exposes more active sites, potentially enhancing catalytic performance compared to that of a more crystalline and ordered phase. The lack of characteristic peaks associated with Fe in the XRD suggests that Fe is incorporated into the α-Ni(OH)_2_ lattice as a dopant (forming a mixed or substituted (Ni,Fe)(OH)_2_) rather than existing as a separate crystalline Fe oxide/hydroxide phase, consistent with previous results.^45^ Under these circumstances, the Fe-related reflections in XRD either overlap with the broader Ni(OH)_2_ peaks or remain below the detection limit due to the low, yet uniform, concentration (see also Figures 1G and S2F).

CeO_2_, Ni_90_Fe_10_/CeO_2_, and Ni_50_Fe_50_/CeO_2_ exhibit BET surface area values of 167.1, 23, and 78 m^2^/g respectively, as evaluated through BET adsorption isotherms (Figure 2B). As we discuss later, this is intriguing since Ni_90_Fe_10_/CeO_2_ exhibits a lower surface area value compared to those of Ni_50_Fe_50_/CeO_2_ and colloidal CeO_2_ even though the OER activity is the highest for Ni_90_Fe_10_/CeO_2_. The isotherm of colloidal CeO_2_ shows a Type IV isotherm, which suggests a mesoporous structure. The reduction of surface area in Ni_90_Fe_10_/CeO_2_ is likely related to NiFe nanoparticles occupying the surface of the support, thus blocking the pores. Another possible reason is the increased tendency of the CeO_2_ nanoparticles to agglomerate in the presence of NiFe nanoparticles, which in turn reduces the available surface area.

**Figure 3:**
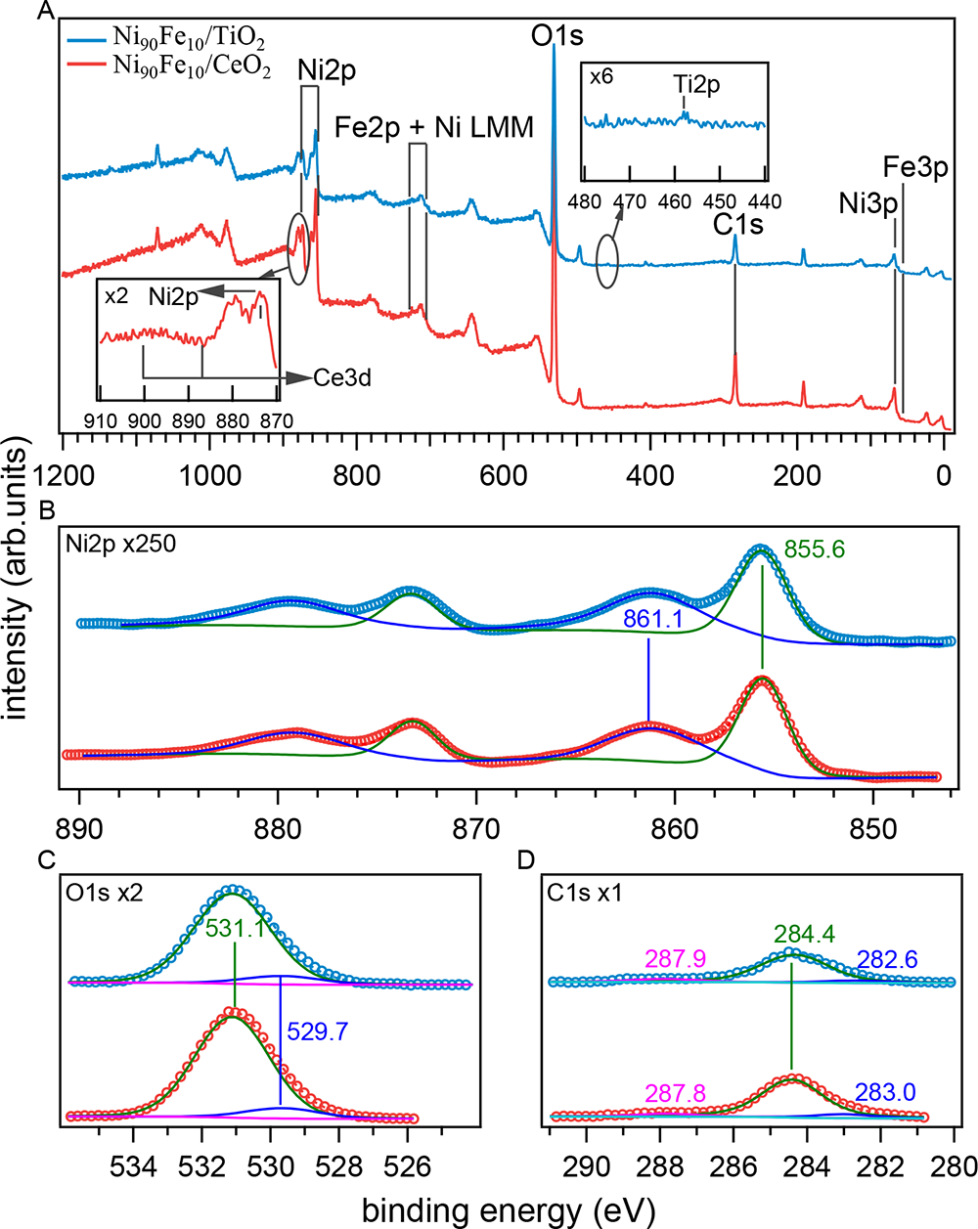
X-ray photoelectron spectra (XPS) of Ni_90_Fe_10_/CeO_2_ and Ni_90_Fe_10_/TiO_2_. (A) Survey spectra of the catalysts. (B) Ni 2p, (C) O 1s, and (D) C 1s high-resolution XPS spectra.

XPS survey spectra of Ni_90_Fe_10_/CeO_2_ and Ni_90_Fe_10_/TiO_2_ reveal prominent peaks corresponding to Ni 2p, O 1s, and C 1s (Figure 3). We attribute the carbon presence to surface contamination due to ambient exposure. The Fe 2p signal, expected around 709 eV, is absent (see also Figure S3), likely due to interference from the Ni LMM Auger peak^46^ suggesting a low Fe 2p intensity. High-resolution XPS spectra of the Ni 2p region (Figure 3B) display two doublets at *E*_*b*_(Ni 2p_3/2_) = 855.6 and 861.1 eV with a spin–orbit separation of Δ*E* = 17.7 eV. These features confirm the presence of Ni^2+^ ions, and therefore in both catalysts Ni is in the oxidized and/or hydroxylated forms.

The XPS spectrum of the O 1s region exhibits only one peak, which can be deconvoluted into two components with *E*_b_ = 529.7 and 531.1 eV (Figure 3C). The minor component at 529.7 eV is typical of the metal–oxygen bond and likely corresponds to oxygen bound to Fe atoms within the catalyst. We also observe that its relative intensity increases significantly for the Ni_75_Fe_25_ sample (Figure S4). The major component at 531.1 eV is associated with the oxygen-containing species adsorbed on the catalyst surface.^47,48^ We note that the samples were not stabilized by calcination. The binding energy values suggest surface hydroxylation or formation of oxyhydroxide species, consistent with the information conveyed by the Ni 2p spectra and the outcome of XRD analysis (Figure 2A). However, the resolution of the present XPS experiments does not permit differentiating between Ni(OH)_2_ and NiOOH species, given the proximity of their characteristic peaks. The C 1s region shows a main peak at 284.4 eV (adventitious carbon) and minor peaks at 283.0/287.8 eV for Ni_90_Fe_10_/CeO_2_ and 282.6/287.9 eV for Ni_90_Fe_10_/TiO_2_ (Figure 3D).^34^ The low-binding energy component corresponds to Fe–C bonds, more prominent in Ni_75_Fe_25_/CeO_2_ (Figure S4D), while the 287.8 eV component indicates oxygen-containing contaminants (C–O, C=O, and COOH) contributing to the 531.1 eV of the O 1s peak.^49^ This component is larger for Fe-rich samples (Figure S4C), suggesting higher reactivity toward environmental contamination.

### Electrochemical Characterization

**Figure 4:**
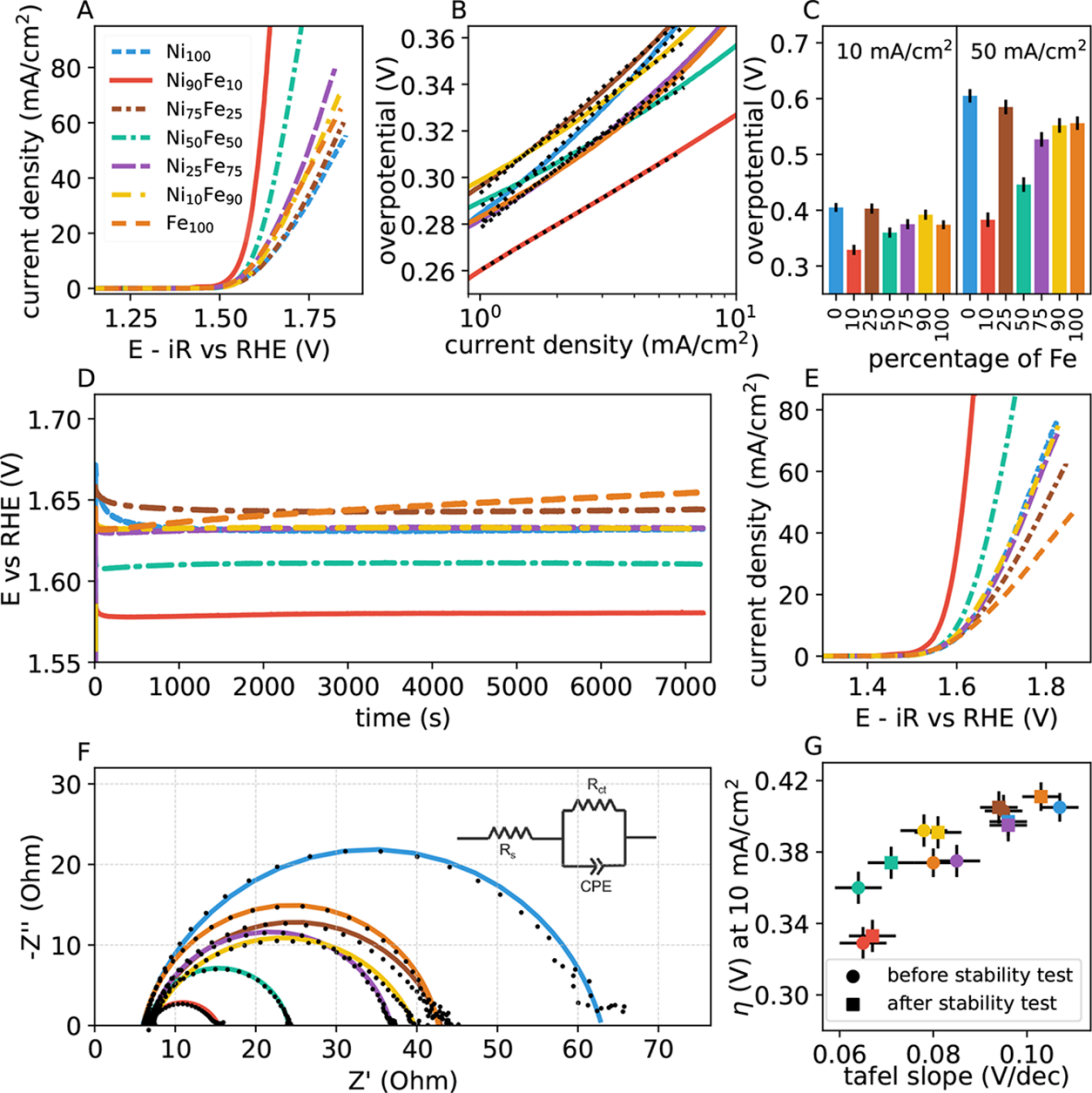
Alkaline oxygen evolution reaction (OER) performance of NiFe catalysts on CeO_2_ support. The color legend for all panels is provided in panel A. (A) OER polarization curve of the catalysts showing that Ni:Fe ratio of 9:1 has the highest activity. (B) Tafel plot of the catalysts. The dotted lines show the corresponding fit to the Tafel equation. (C) Overpotential values at 10 mA/cm^2^ (left) and 50 mA/cm^2^ (right) current densities. (D) Chronopotentiometry measurements (stability tests). (E) Linear sweep voltammetry (LSV) measurements following the stability test. (F) Electrochemical impedance spectroscopy (EIS) measurements showing that Ni:Fe ratio of 9:1 has the lowest charge transfer resistance. The fit (solid line) is according to the Randles-like circuit shown in the inset. (G) Overpotential vs Tafel slope before (circles) and after (squares) the stability test.

We start the electrochemical analysis by fixing the catalyst support (CeO_2_) and varying the Ni:Fe ratio to evaluate how the catalytic activity changes as a function of the iron content. The polarization curves obtained through linear sweep voltammetry (LSV) show that there is significant variation in the OER activity as we change the Ni:Fe ratio (Figure 4A). Ni_90_Fe_10_/CeO_2_ catalyst achieves the benchmark 10 mA/cm^2^ current density (where electrode disk area is used) at a potential of 1.57 V which is much lower than the corresponding potential of 1.65 V for Ni_100_/CeO_2_, highlighting the impact of Fe incorporation. For these experiments, we use the Aemion+ ionomer, a choice motivated by our focus on AEM electrolyzers. Aemion+ is shown to inhibit the Ni^2+^ to Ni^3+^ in Ni-based catalysts and hence suppress the catalytic performance.^30^ Still, at a loading of 0.4 mg/cm^2^, Ni_90_Fe_10_/CeO_2_ achieves a 10 mA/cm^2^ current density at an overpotential of 329 mV. This is lower than the overpotential of previously reported NiFe OER catalysts using Aemion+,^30^ showing that CeO_2_ enhances the catalytic performance. Figure 4C shows the overpotential of the catalysts at 10 and 50 mA/cm^2^ current densities. Ni_90_Fe_10_/CeO_2_ shows a Tafel slope of 0.065 V/decade which is the lowest Tafel slope among the tested catalysts (see Figure 4B). The lower Tafel slope indicates easier electron transport and favorable reaction kinetics for OER at high current densities.^50−52^

The electrode kinetics of the NiFe/CeO_2_ catalysts can be assessed through electrochemical impedance spectroscopy (EIS) measurements (Figure 4F). The Nyquist plots are fitted with a Randles-like circuit model, using Python package *impedance*,^53^ with constant phase elements to obtain the solution resistance (*R*_s_) and charge transfer resistance (*R*_ct_). A lower *R*_ct_ value which is obtained from the diameter of the semicircle in the Nyquist plot means rapid charge transfer kinetics, which is favorable for a good OER catalyst. Ni_90_Fe_10_/CeO_2_ exhibits a lower charge transfer resistance of 8.87 Ω compared to other Ni:Fe ratios as shown in Figures 4F and S6. Also, the OER activity of all the NiFe catalysts compares favorably with most of the NiFe-based catalysts reported in the literature.^54^

As we discussed before, from BET measurements, Ni_90_Fe_10_/CeO_2_ has a lower surface area (23 m^2^/g) compared to Ni_50_Fe_50_/CeO_2_ (78 m^2^/g). This suggests that the superior activity of the Ni_90_Fe_10_/CeO_2_ toward OER does not come from the enhanced surface area but from the intrinsic catalytic property of the active sites of the Ni_90_Fe_10_/CeO_2_ catalyst. In other words, the quality of the active sites overtakes the quantity of active sites in the evaluated catalysts. This points to the correlation between the electronic structure modification and the OER activity in alkaline media. It is known that when the Ni:Fe ratio changes, the charge transfer effect causes the mutual modulation of the metal atoms.^55^ This tuning influences the active sites and reactant interactions, thus reducing the adsorption energy of reactants, the formation energy of intermediates, and the desorption energy of products. Hence the overall catalytic performance is augmented.^27^ This leads us to conclude that the electronic structure variation due to the varying Ni:Fe ratio is one reason for the differential OER performance of the catalysts. Thus, although the BET isotherms reveal that there is a decrease in the surface area of CeO_2_ with Ni and Fe addition, the lower surface area of Ni_90_Fe_10_/CeO_2_ does not hinder the catalytic activity, suggesting that the optimized Ni–Fe composition and electronic interactions are the primary factors driving its superior OER activity.

The stability of the OER catalysts plays an important role in large-scale electrolysis. All of the NiFe catalysts exhibit a slight increase in the overpotential after the chronopotentiometry measurements, but Ni_90_Fe_10_/CeO_2_ shows the least change over time (Figure 4D). LSV measurements after the stability test reveal that for Ni_90_Fe_10_/CeO_2_ the overpotential at 10 mA/cm^2^ only shows a slight change, in sharp contrast to the other NiFe catalysts (Figure 4E,G).

**Figure 5:**
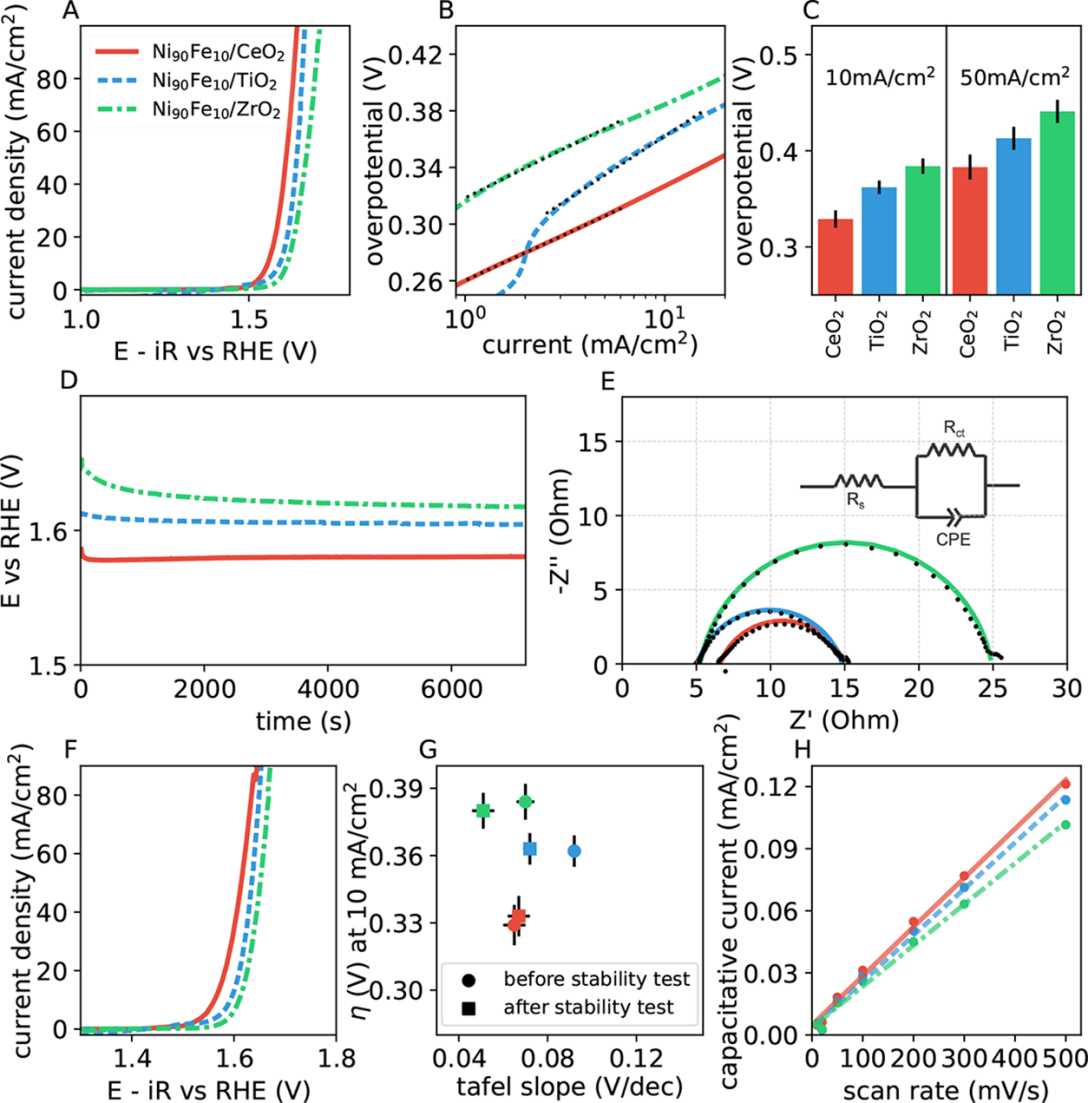
Alkaline oxygen evolution reaction (OER) performance of Ni_90_Fe_10_ catalysts on CeO_2_, TiO_2_, and ZrO_2_ supports. The color legend for all panels is provided in panel A. (A) Linear sweep voltammetry (LSV) measurements of the catalysts showing that CeO_2_ is the best-performing support. (B) Tafel plot of the catalysts. The dotted lines show the corresponding fit to the Tafel equation. (C) Overpotential values at 10 mA/cm^2^ (left) and 50 mA/cm^2^ (right) current densities. (D) Chronopotentiometry measurements (stability tests). (E) Electrochemical impedance spectroscopy (EIS) measurements. The fit (solid line) is according to the Randles-like circuit shown in the inset. (F) LSV measurements following the stability test. (G) Overpotential vs Tafel slope before (circles) and after (squares) the stability test. (H) Double-layer capacitance measurements.

Let us now evaluate the impact of the catalyst support by keeping the Ni:Fe ratio constant at 9:1 and by comparing the performance of the catalysts between CeO_2_, TiO_2_, and ZrO_2_. The weight percent of the support is kept constant at 30. Our results show that CeO_2_ has a lower overpotential than the other two supports (Figure 5A,C). The overpotential at 10 mA/cm^2^ of Ni_90_Fe_10_/CeO_2_ is 329 mV compared to 362 mV for Ni_90_Fe_10_/TiO_2_ and 384 mV for Ni_90_Fe_10_/ZrO_2_. However, it is interesting to note that all three supports show good performance, with relatively small differences between them. Figure 5B shows the Tafel slopes of the catalysts, and Ni_90_Fe_10_/CeO_2_ has a lower Tafel slope of 65 mV/dec compared to 73 mV/dec for Ni_90_Fe_10_/TiO_2_ and 72 mV/dec for Ni_90_Fe_10_/ZrO_2_. The charge transfer resistance obtained from Nyquist plots of Ni_90_Fe_10_/CeO_2_ is 8.36 Ω which is lower than for Ni_90_Fe_10_/TiO_2_ and Ni_90_Fe_10_/ZrO_2_ (Figure 5E). Chronopotentiometry measurements show that all three catalysts are stable under operation (Figure 5D). Intriguingly, Ni_90_Fe_10_/TiO_2_ and Ni_90_Fe_10_/ZrO_2_ show a slight increase in performance with time, indicating potential surface reconstructions improving the activity of these catalysts (Figure 5F,G).

**Figure 6:**
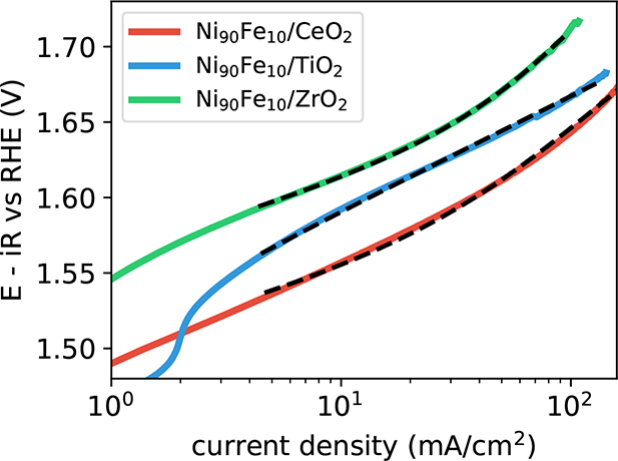
Polarization curves of Ni_90_Fe_10_ catalysts on CeO_2_, TiO_2_ and ZrO_2_ supports and the corresponding fit using eq 1.

To understand the reason for the differences in the OER activity between different supports, we conduct double-layer capacitance (*C*_dl_) measurements to assess the relative electrochemical surface area (ECSA)^56^ (Figure 5H). Ni_90_Fe_10_/CeO_2_ has a higher double-layer capacitance compared to those of Ni_90_Fe_10_/TiO_2_ and Ni_90_Fe_10_/ZrO_2_, and in turn, has a higher electrochemical surface area. In addition to the higher ECSA, the better initial performance of CeO_2_ can also be attributed to better metal–support interactions.^57^ It is known that the CeO_2_/Ni(OH)_2_ heterojunction enhances the generation and reconstruction of NiOOH active sites.^58^ CeO_2_ has superior and fast redox properties as it can transform between Ce^3+^ and Ce^4+^ oxidation states which is favorable for the electrochemical reaction^59^ This suggests that CeO_2_ may exist in a partially reduced state containing Ce^3+^ and Ce^4+^ oxidation states and oxygen vacancies, further corroborated through the lattice distortions observed in HR-TEM (Figure 1D). Also, it is known that oxygen vacancies improve the ionic conductivity and catalytic performance. This is consistent with the reported observation of higher oxygen storage capacity and good ionic conductivity for CeO_2_.^31^ A surface CeO_2_ layer has been shown to increase the OER stability in NiFeO_*x*_ catalysts as the surface layer prevents Fe dissolution in the electrolyte.^31^ The Ni–Fe–CeO_2_ electronic modulation could also improve the activity of the intrinsic active sites for the OER reaction in the catalysts.^57^ In comparison, although TiO_2_ has insufficient electrical conductivity,^60,61^ it has high corrosion resistance and ultrahigh stability in addition to strong metal–support interactions.^28^ This potentially explains the better stability of the catalyst on the TiO_2_ support. At this point, it is worthwhile to closely examine the reaction pathway at work here. Assuming that the reaction proceeds as per an electrochemical oxide path given by
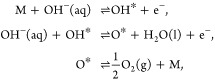
following the calculations in ref (62), the current density *j* obeys the equation:
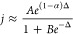
1Here, *A* and *B* are constants, α is the charge transfer coefficient,  is the dimensionless potential, *F* is Faraday’s constant, *R* is the universal gas constant, and *T* is the temperature. As shown in Figure 6, we fit the polarization curves to eq 1, showing excellent agreement, suggesting that the reaction indeed proceeds through the electrochemical oxide path for all three supports.

## Conclusions

It has long been known that Ni doped with a small amount of Fe acts as a highly efficient electrocatalyst for the OER reaction. The present study confirms that this holds true for NiFe catalysts on metal oxide supports in the presence of an anion exchange ionomer as well. Increasing the Fe content in our system leads to complex changes in catalytic performance, with multiple local minima in the overpotential, indicating that the electronic interactions between Ni and Fe are highly complex. All three supports that we investigate, CeO_2_, TiO_2_, and ZrO_2_, show high activity and could potentially be utilized for commercial purposes. The slightly better performance of CeO_2_ could be attributed to comparatively high electronic conductivity, oxygen vacancy formation, and redox flexibility than TiO_2_ and ZrO_2_, which collectively enhance the electron transfer processes essential for OER.^31,63^ However, TiO_2_ has better stability when compared to CeO_2_ suggesting that it stabilizes the metal active sites and resists degradation under electrochemical conditions. To understand the stability and durability of these catalysts under practical conditions, it is necessary to conduct AEM cell tests, which represent an important direction for future research. The reported high activity and stability of the Ni_90_Fe_10_ on CeO_2_, TiO_2_, and ZrO_2_ catalysts make them promising nonprecious metal catalysts for application in AEM electrolyzers.

## Data Availability

All data used to generate the figures in this manuscript as well as the corresponding analysis and plot scripts are available at https://github.com/neethukvarghese/nife-mo-alkaline-oer-data.

## Supporting Information Available

Table showing FAAS measurements of NiFe/CeO_2_; additional SEM images of NiFe/CeO_2_; additional TEM images of Ni_90_Fe_10_/CeO_2_; XPS spectra of Ni_75_Fe_25_/CeO_2_; Fe 2p and Ni 3p-Fe 3p spectra of Ni_90_Fe_10_/CeO_2_, Ni_90_Fe_10_/TiO_2_, and Ni_75_Fe_25_/CeO_2_; overpotential, Tafel slope, and charge transfer resistance as a function of Fe content; CV plots of Ni_90_Fe_10_/CeO_2_, Ni_90_Fe_10_/TiO_2_, and Ni_90_Fe_10_/ZrO_2_ and details of uncertainty analysis (PDF)
