# Supplementary material for: NiFe on CeO2, TiO2, and ZrO2 Supports as Efficient Oxygen Evolution Reaction Catalysts in Alkaline Media
Source: ACS Appl Energy Mater. 2025 Feb 24;8(5):3087–95. doi: 10.1021/acsaem.4c03268 (PMC11902787; doi:10.1021/acsaem.4c03268)
Supplement: Supplementary file 1 — ae4c03268_si_001.pdf [file ae4c03268_si_001.pdf]

Supporting Information: NiFe on CeO_2, TiO_2 and ZrO_2 Supports as Efficient Oxygen Evolution Reaction Catalysts in Alkaline Media

Neethu Kochukunnel Varghese,*,† Elina Mkrtchian,‡ Anshika Singh,¶ Letizia Savio,‡ Massimiliano Boccia,§ Vincenza Marzocchi,§ and Antonio Comite*,†

†*Department of Chemistry and Industrial Chemistry, University of Genoa, 16146 Genoa, Italy*

‡*IMEM-CNR UOS Genoa, 16146 Genoa, Italy*

¶*Department of Physics, University of Genoa, 16146 Genoa, Italy*

§*H2 Energy SRL, 26026 Pizzighettone, Cremona, Italy*

E-mail: neethu.kochukunnel.varghese@edu.unige.it; antonio.comite@unige.it

Table S1: Concentration of Ni and Fe in the 20 mg/L catalyst solution calculated using FAAS.

Sample	Conc.of Ni(mg/L)	Conc.of Fe (mg/L)	Ni:Fe ratio
Ni_{100}/CeO_2	6.86 ± 0.08	0	1 : 0
$Ni_{90}Fe_{10}/CeO_2$	5.62 ± 0.04	0.7 ± 0.05	0.89 : 0.11
$Ni_{75}Fe_{25}/CeO_2$	3.99 ± 0.03	1.2 ± 0.07	0.76 : 0.24
$Ni_{50}Fe_{50}/CeO_2$	2.51 ± 0.05	2.1 ± 0.04	0.54 : 0.46
$Ni_{25}Fe_{75}/CeO_2$	1.35 ± 0.04	3.2 ± 0.04	0.29 : 0.71
$Ni_{10}Fe_{90}/CeO_2$	0.61 ± 0.03	3.6 ± 0.07	0.14 : 0.86
Fe_{100}/CeO_2	0	4 ± 0.07	0 : 1

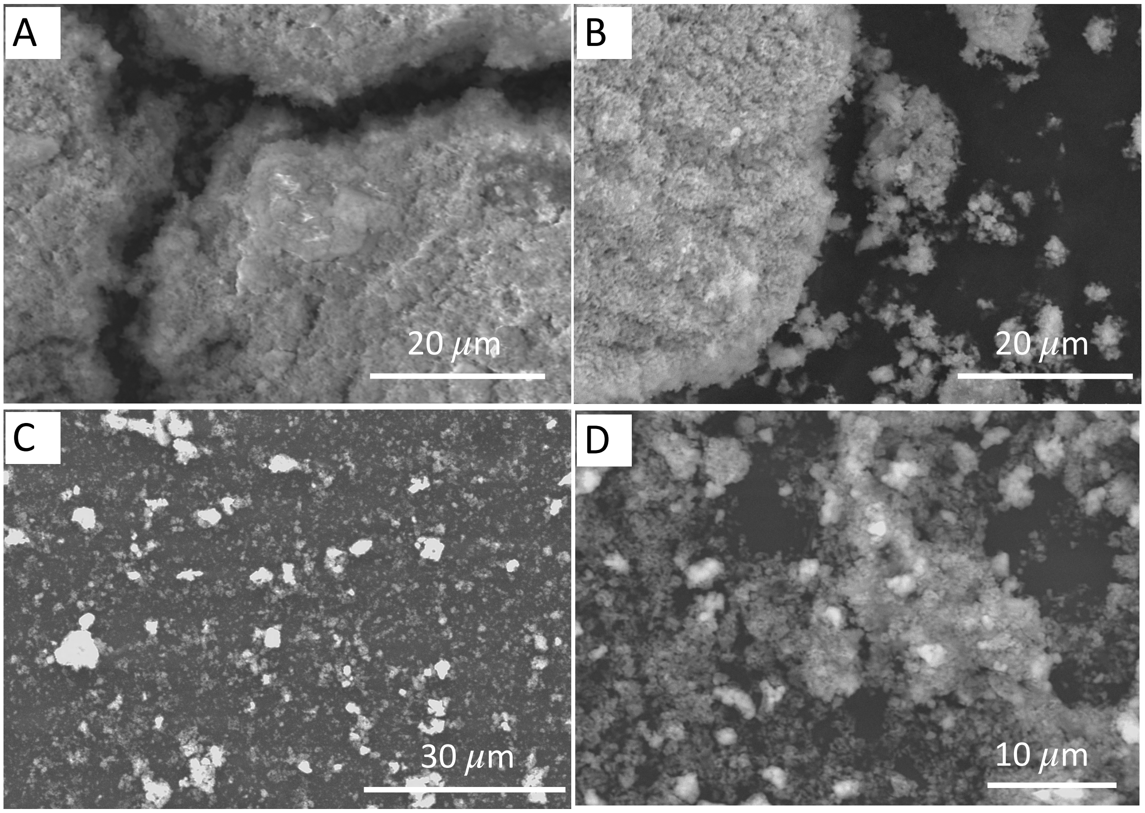

Figure S1: SEM images of A) Ni_{100}/CeO_2, B) $Ni_{90}Fe_{10}/CeO_2$, C) $Ni_{75}Fe_{25}/CeO_2$ and D) $Ni_{50}Fe_{50}/CeO_2$.

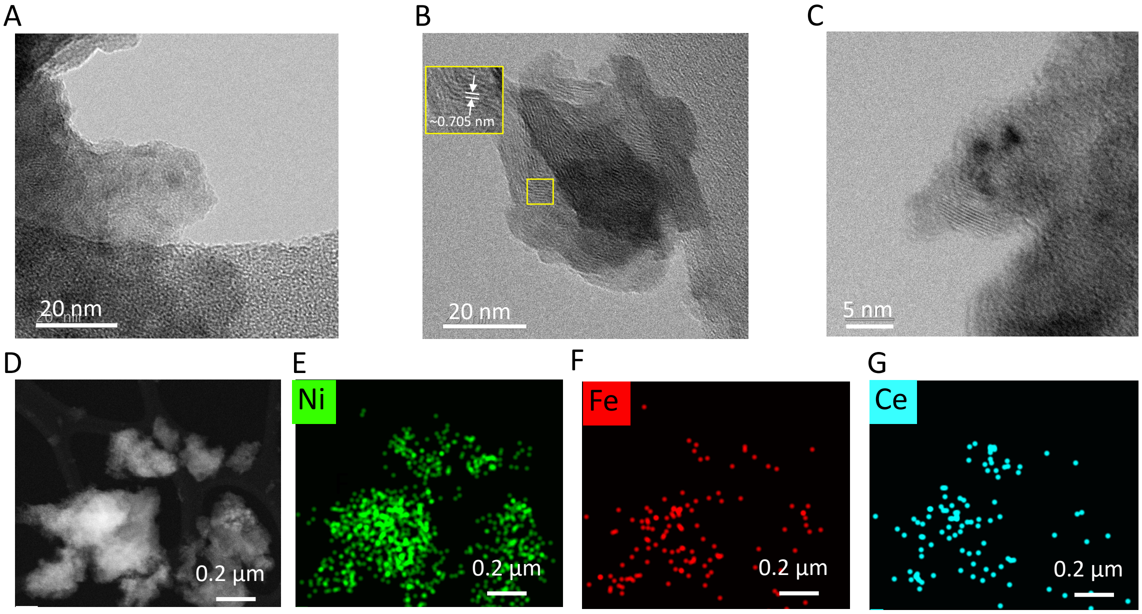

Figure S2: A-C) TEM images of $Ni_{90}Fe_{10}/CeO_2$. D-G) TEM EDS mapping of Ni, Fe and Ce.

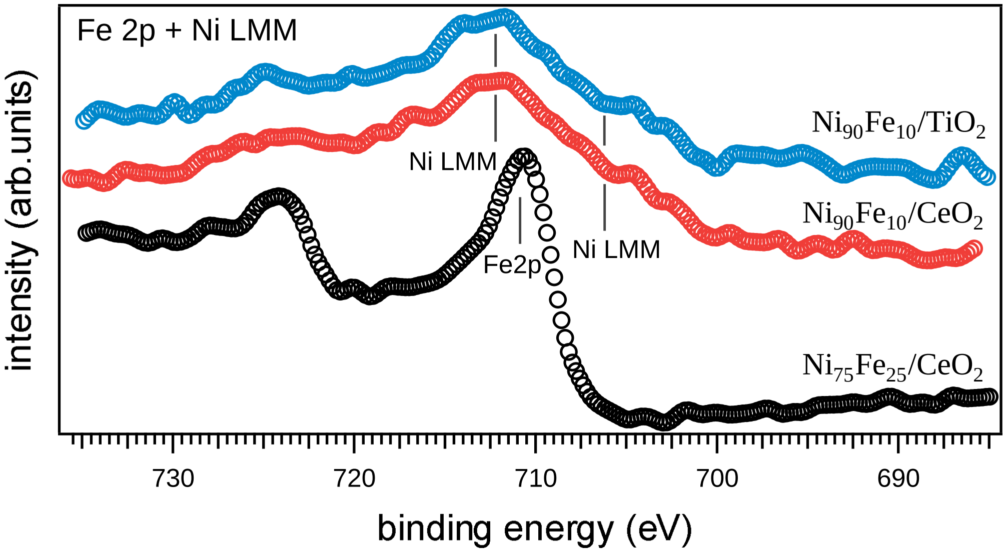

Figure S3: A) High-resolution XPS spectra of the Fe 2p and Ni LMM Auger peaks. Note that no separate Fe 2p peak is visible for the $Ni_{90}Fe_{10}$ catalysts, likely due to its low intensity compared with the Ni LMM Auger peak. However, the Fe 2p peak is visible for the $Ni_{75}Fe_{25}$ catalyst.

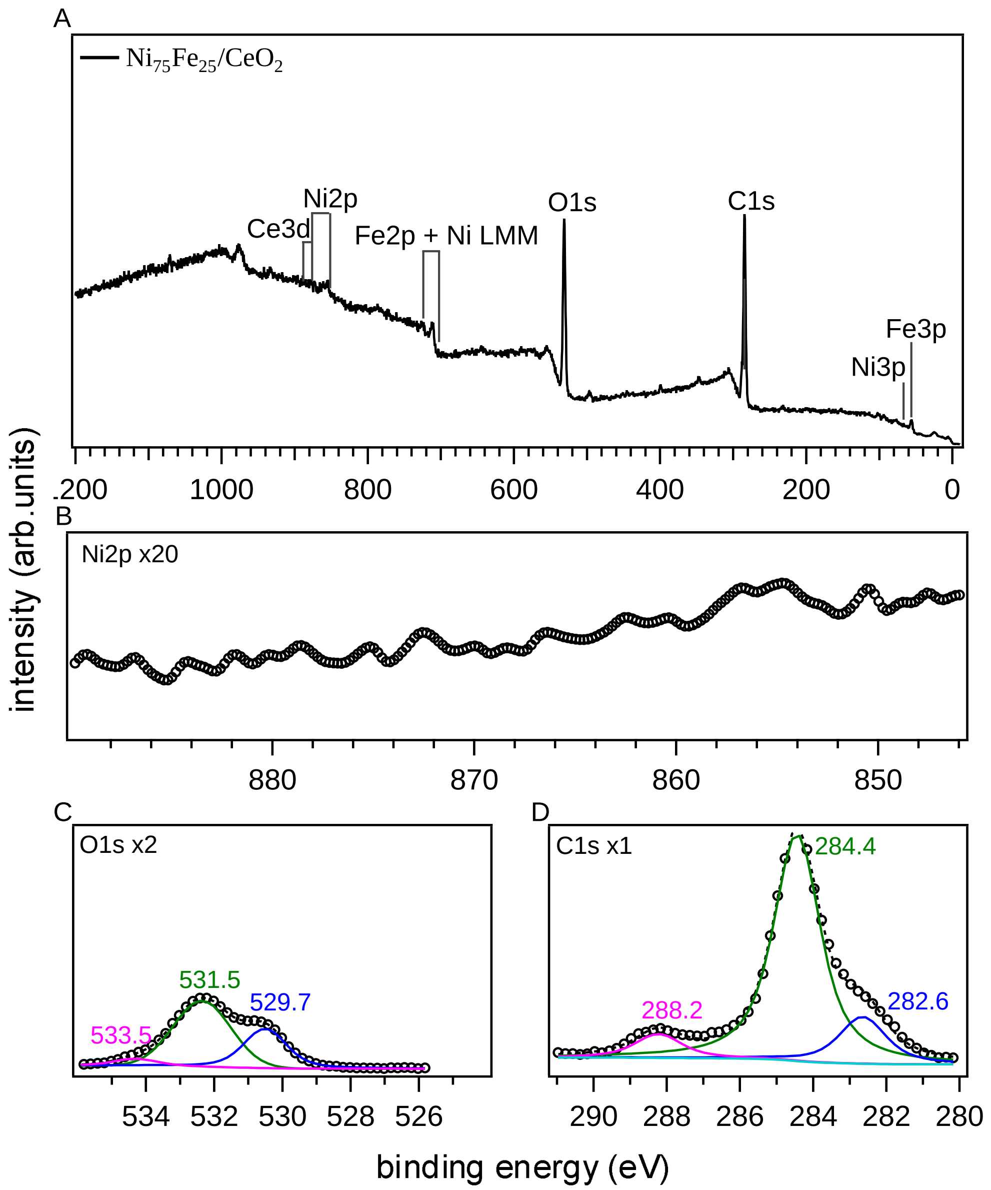

Figure S4: XPS spectrum of $Ni_{75}Fe_{25}/CeO_2$. A) Survey spectrum of the catalyst. B), C) and D) High-resolution spectra of the Ni 2p, O 1s and C 1s regions, respectively. It is interesting to note that the Ni 2p signal is weak despite Ni being the major constituent of the catalyst.

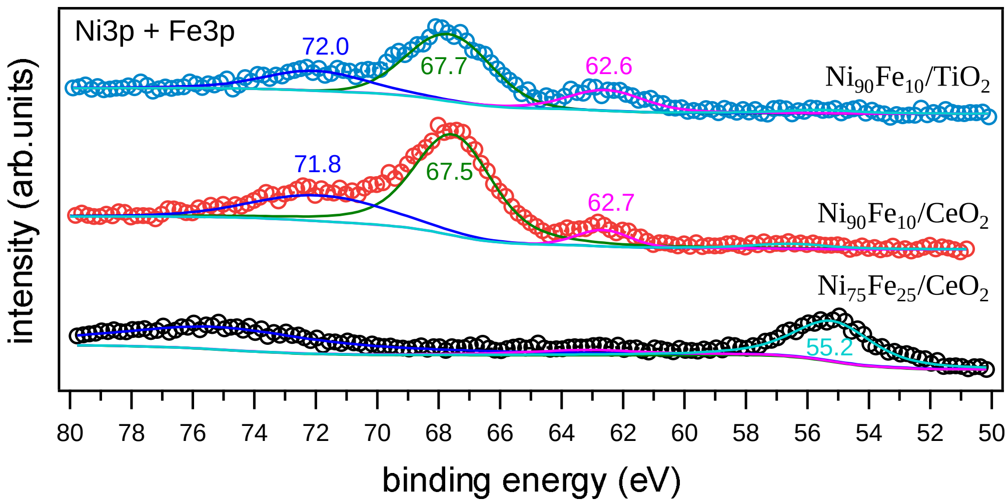

Figure S5: High-resolution XPS spectra of the Ni 3p and Fe 3p regions. $Ni_{90}Fe_{10}$ spectra are dominated by the Ni 3p signal at around 67.5 eV and by it is satellite at around 71.7 eV. In contrast, Fe 3p signal appears at 55.2 eV only for the $Ni_{75}Fe_{25}$ sample. The peak at 62.6/62.7 eV corresponds to the Na 2s line and indicates the presence of Na from the synthesis ($Ni_{90}Fe_{10}/CeO_2$ and $Ni_{90}Fe_{10}/TiO_2$).

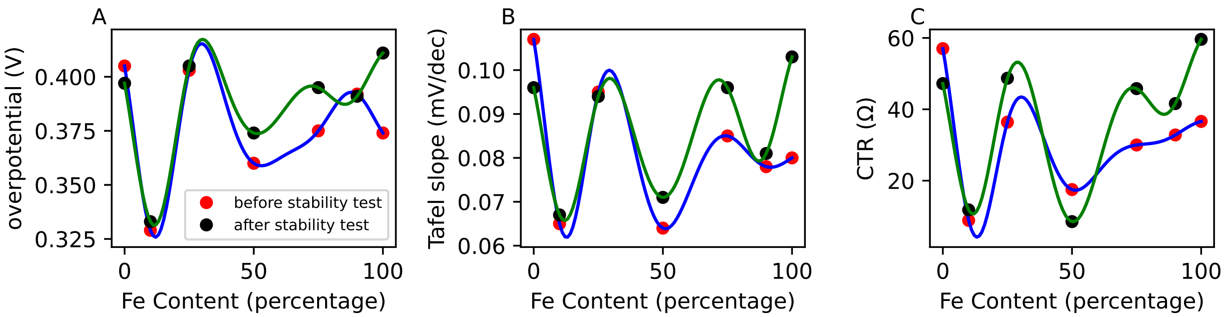

Figure S6: A) Overpotential as a function of the percentage of iron content before and after the stability test. The fit is with a Gaussian process regressor. B) and C) The corresponding Tafel slope and charge transfer resistance plots.

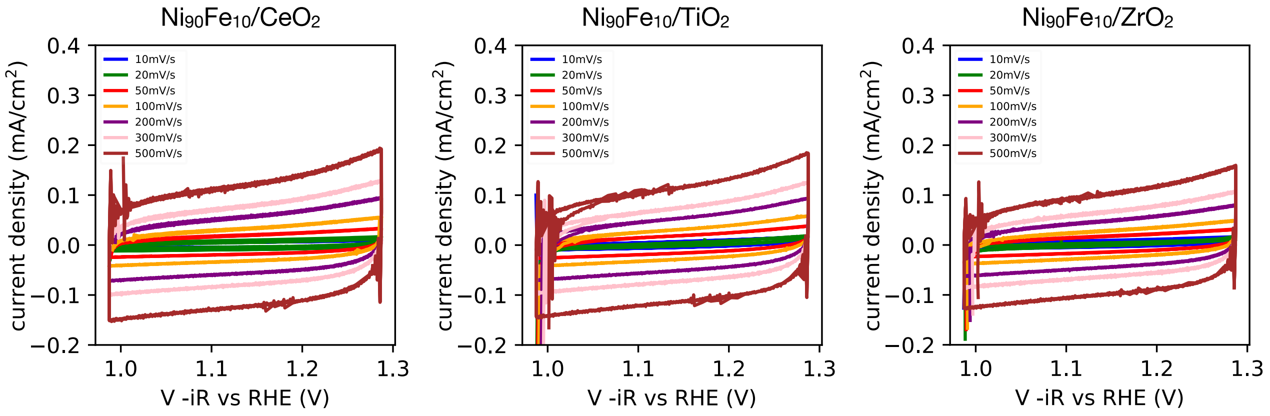

Figure S7: Cyclic voltammograms in the non-Faradaic region for double layer capacitance measurements shown in Fig. 5 (g).

Uncertainty analysis

Overpotential calculation: The error in the estimation of overpotential value was computed through error propagation given by:

$$\sigma_\eta = \sqrt{\sigma_V^2 + (-I\sigma_{R_s})^2 + (R_s\sigma_I)^2},$$

where σ_V is the error in the Voltage measurement, I the current, R_s is the solution resistance estimated by fitting the EIS curves, σ_I is the error in the current measurement and σ_{R_s} is the error in the calculation of the solution resistance.

Tafel slope calculation: The uncertainty in Tafel slope calculation was obtained from the error in the fitting of the Tafel curve, after accounting for the error in the overpotential measurement. For more details, please refer to the code published with this manuscript.

FAAS measurements: Three independent measurements were conducted for each of the samples, and the reported value represents the mean. The error was estimated based on the range of the measurements.
